# Surgery

**Published:** 1876-07

**Authors:** 


### Summary of Progress in the Medical Sciences.

### I. Surgery.

1. *Uniting Tendons by Suture.* Fillauz. (*The Medical and Surgical Reporter*, March 18, 1876.)

A laborer, fifty-four years old, sustained a lacerated wound on the dorsal aspect of the right hand. The extensor tendons of the fourth and fifth fingers were torn through. The wound healed in due time, but these fingers could not be extended actively. Two months after the injury, Fillauz, employing local anæsthesia, easily succeeded in finding the peripheral tendinous stumps, but the proximal stumps had retracted under the dorso-carpal ligament. The operator, therefore, dissected the tendon of the middle finger in which he made a longitudinal incision, through which the freshened stumps of the tendons were passed and fixed by wire suture.

The wounds having healed, the patient could extend all his fingers, though there was firm adhesion between the skin and the cicatrix of the tendon.

Reference is made to a second successful case.

2. *Treatment of Carbuncular Disease in Man by the Subcutaneous Injection of Anti-Virulent Fluids.* Dr. L. H. Raimbert. Translation by Dr. Wm. C. Dabney, M.D. (*Virginia Med. Monthly*, March 1, 1876.)

Raimbert tried the subcutaneous injection of carbolic acid, one part to fifty, in two cases of carbuncle, after other means had failed, and in one case the hypodermic injection of iodine, one part to five hundred, with favorable results. In the first case he was consulted on account of a malignant œdema of the face, by a farmer who, a week before, had lost a number of sheep with inflammation of the spleen. The disease first appeared as a number of small pustules behind the right ear, followed by a considerable infiltration of the cheeks, lips and structures beneath the jaw. On the third day, the white-hot iron was applied around the diseased part, a distance from it of from four to six centimetres, and the surface of the scabs was sprinkled with corrosive sublimate. On the following day, as soon as the swelling had diminished, Raimbert injected ten or twelve syringefuls of a two per cent. solution of carbolic acid into the whole extent of the cheek. The result was astonishing. The injections were repeated on the following day. The healing progressed uninterruptedly.

The second case was that of a farmer who, in opening a vein in a cow suffering from gangrenous inflammation of the spleen, had inflicted a slight abrasion of the skin of the left ring finger. Three days afterwards, he noticed at the point of abrasion a small papilla depressed in the centre, which the physician consulted declared was malignant pustule, and applied caustic potash. On the following day, there was considerable increase in extent and severity, and the swelling had been decidedly augmented by the use of the caustic. The swelling rapidly extended to the whole arm, which became so hard and tense that it was necessary to make incisions in both arm and forearm to lessen the tension.

On the fourth day, Raimbert saw the patient, and, mindful of the result in his former case, he injected nearly forty syringefuls of carbolic acid solution (two per cent.) in the whole length of the arm. The result was "brilliant."

The following day the arm was less hard ; and the injections were repeated on this day, morning and evening.

From that time the improvement progressed slowly and steadily, so that at the end of two months after, the gangrenous part of the ring finger, a part of the back of the hand, and some parts which had become gangrenous at the seat of the puncture, had been cast of. All the wounds were found to be well healed.

In a third case, where the wife of a tradesman had been handling hides, the affection began as a small knob on the right cheek, the connective tissue around which had become infiltrated for a considerable distance, and from which a red band of lymph vessels ran directly to an enlarged gland behind the jaw. After removing the skin at the centre, the wound thus made was treated with corrosive sublimate.

The swelling had greatly increased on the following day, and was progressing towards the neck and eyelids; that evening, 4½ syringefuls of a solution of iodine, one part to 500, were injected under the skin of the cheek and lower jaw. On the following day no change having occurred in the swelling, the injection was repeated, and on the next day there was a manifest diminution in the size of the part. The injection was again repeated, with marked and progressive improvement.

3. *Epithelioma of the Tongue and its Relation to Psoriasis of this Organ.* Communication of M. Trélat to the Surgical Society. (*La Tribune Médicale*, December, 1875.) Translation by Wm. C. Dabney, M.D. (*Virginia Med. Monthly*, March, 1876.)

M. Trélat presented three cases illustrating a close relation between psoriasis of the tongue and subsequent lingual epithelioma.

In the first case the cancerous affection could not be arrested by surgical means. The patient rapidly succumbed to the well known complications of malignant affections of the organ in question. Very slight lingual psoriasis preceded the outbreak of cancer. In the second and third cases the relation of the two affections is as close as in the first.

4. *New Rhinoplastic Operation.* (*St. Louis Clinical Record*, March, 1876.)

Upon the authority of *L'Union Méd. du Canada*, the *Gazette Médicale de Paris* mentions a case under the care of M. Hardie where the skin of a finger was borrowed to furnish the soft parts for a new nose; and the phalangeal bone was utilized to form the solid portion. The patient was a girl aged 16 years. The alæ and a portion of the septum nasi remained. The improved nose made a good feature.

5. *Introductory at the Pennsylvania Hospital on Bonwill's Method of Inducing Anæsthesia.* ADDINELL HEWSON, M.D. (*Philadelphia Med. Times*, March 4, 1876.)

After a casual reference to the value of chloroform and ether in robbing surgery of the greatest of its terrors, the lecturer remarked that we are in want of measures to diminish the sensitiveness of a part, so that patients can be handled or examined with as much force as may be necessary to detect all the phenomena in their cases. In the most serious cases for manipulative examinations we have not heretofore hesitated to use ether and chloroform, but in many of them we should feel that we were in want of some means to be used without inconvenience or annoyance. This desideratum, Dr. Hewson believes is found in Dr. M. G. A. Bonwill's method of diminishing or allaying sensibility by rapid respirations. The confusion of sight and bewilderment of mind induced by a rapid and deep respiration after violent running, or blowing hard to ignite a fire, Dr. Bonwill associated with numbness of sentient nerves. Pursuing the subject, he has brought it to practical use in his profession, that of dentistry, in which he uses it constantly to diminish the sensitiveness of dentine, and even to produce such insensibility as to allow the extraction of a molar tooth without pain.

Dr. H. has employed the method in stitching wounds, in handling over-sensitive parts, in probings, and the like. The lecturer failed before the class in two cases in which he attempted to use Bonwill's method. The patients were both boys; neither would continue inspirations anything like long enough, three minutes being required. The want of success was due to great emotional excitement and possibly from the dread of the introduction of the sounding-board to be used in conjunction with the probe in the search for dead bone. Dr. Bradford, the reporter of the lecture, then volunteered to try the process before the class. It was his first attempt, and was made sitting erect, with his right hand resting upon the table. Breathing rapidly for about three minutes, was attended first with a tingling sensation of the surface, especially of the fingers, and a feeling as though the surface was swelling. Then there followed a dizziness or confusion in the head, with consciousness well preserved, but with a feeling of inability to resist or act in an independent way. He remembered well, being frequently asked by the doctor if he was hurting him, but had no recollection afterwards of the pin sticking him, much less of its having been firmly imbedded in his flesh, as he found it when he had ceased the rapid respirations and the anæsthetic effect had passed off.

6. *Amputation in a Diabetic Patient; Sudden Death.* VERNEUIL. (*Le Progrès Méd.* No. 22.)

A railroad employé, 52 years old, had his left leg crushed by a car. He was of robust and vigorous frame, in good condition for operative interference, and the indications for removal of the limb were clear.

The operation was accompanied by a rather abundant hæmorrhage, but the next night was passed comfortably and no traumatic fever followed.

The patient enjoyed perfect quiet until 10 o'clock of the day after, when a singular and rapid change occurred in his condition. He was found in the dorsal decubitus, with head thrown back, the eyes half closed, the features drawn, brown sordes about the teeth, a peculiar fetor of the breath, a yellowish hue of the skin contrasting singularly with the injection of the capillaries, cold and cyanosed extremities, oppression, subdelirium, incoherency, and extreme, incessant agitation.

It was then discovered that he had been long affected with diabetes mellitus; his urine was found to contain 35 per cent. of sugar; specific gravity, 1030. The temperature was soon, 37° C.; pulse, 140; respirations, 32. Death ensued during the night.

Post-mortem, the stump was found in an excellent condition. The cortical substance of the kidneys was double the normal volume, with prolongations into the pelves, separating the medullary substance into islets. There was some thickening of the capsules, with interspersed patches of a white color. Cretaceous tubercles were seen at the pulmonary apices, the lower lobes of the lungs being red, œdematous and friable. The liver was enlarged, reddish and granular, as in cirrhosis, but not indurated. The spleen was softened and pulpy under the fingers. Under the microscope, a granulo-fatty degeneration was discovered in the liver, kidneys and testicles. (The latter fact explains the impotence and aspermatism of diabetes.)

Verneuil justly concludes that operations should not be performed upon persons affected with organic visceral disease, except in cases where the lesions demanding operative interference are incurable by natural processes.

